# Attention to pulmonary arteriovenous fistula in a case of transient hypoxemia and cerebral infarction during pregnancy: a case report and literature review

**DOI:** 10.1186/s12884-023-05946-2

**Published:** 2023-08-31

**Authors:** Lijuan Shu, Linli Luo, Yunxia Zuo

**Affiliations:** 1 Department of Anesthesiology, West China Hospital, Sichuan University, Chengdu, 610041 Sichuan P.R. China; 2 The Research Units of West China (2018RU012) - Chinese Academy of Medical Sciences, West China Hospital, Sichuan University, Chengdu, 610041 Sichuan P.R. China; 3 Key Laboratory of Birth Defects and Related Diseases of Women and Children (Sichuan University), Ministry of Education, Chengdu, 610041 Sichuan P.R. China; 4 Department of Obstetrics and Gynecology Intensive Care Unit, West China Second University Hospital, Sichuan University, Chengdu, 610041 Sichuan P.R. China; 5 Department of Anesthesiology, West China Second University Hospital, Sichuan University, Chengdu, 610041 Sichuan P.R. China

**Keywords:** Hypoxemia, Cerebral infarction, Pulmonary arteriovenous fistula, Case report

## Abstract

**Background:**

Pulmonary arteriovenous fistula is rare during pregnancy. Pulmonary arteriovenous fistula presents no pulmonary symptoms in most patients but can be exacerbated by pregnancy. If not diagnosed and treated promptly, pulmonary arteriovenous fistula can lead to respiratory failure, stroke, spontaneous hemothorax, or other fatal complications.

**Case presentation:**

A 29-year-old healthy pregnant woman presented with a transient drop in blood oxygen level of unknown cause during a routine examination at 34 weeks of gestation and during a cesarean section at 38 weeks of pregnancy. The patient’s oxygen saturation quickly returned to normal and was not further investigated. On day 3 postpartum, the patient suddenly displayed slurred speech and right limb myasthenia. A head magnetic resonance imaging revealed cerebral infarction in the left basal ganglia. Subsequent computed tomography pulmonary arteriography revealed bilateral pulmonary arteriovenous fistula, which was likely the cause of cerebral infarction. The patient was transferred to the Department of Thoracic Surgery after one month of treatment and successfully underwent percutaneous embolization of pulmonary arteriovenous fistula.

**Conclusion:**

Pulmonary arteriovenous fistula should not be neglected if a pregnant woman presents with transient hypoxemia and cerebral infarction. A transient decrease in pulse oxygen saturation that cannot be explained by common clinical causes can be an early warning sign of the disease. Early diagnosis and multidisciplinary management could improve the prognosis.

## Background

Pulmonary arteriovenous fistula (PAVF) is a rare pulmonary vascular malformation that allows direct communication between the pulmonary artery and vein, bypassing the capillaries [1]. PAVF is mainly congenital but can be acquired in some cases, such as in patients with cirrhosis or those undergoing surgery for congenital heart disease. PAVF has been reported in 0.02‒0.03% of the population, primarily in women, and has a mortality rate of up to 11% [2]. Approximately 70% of PAVF cases are identified in patients with hereditary hemorrhagic telangiectasia (HHT), while the remaining 30% are sporadic cases unrelated to HHT [3]. Although PAVF has rarely been reported during pregnancy, medical screening in recent years has revealed a ≥ 0.38% PAVF incidence in the general asymptomatic population [4]. Therefore, more PAVF cases may be encountered during pregnancy.

Pregnancy has been reported to be a risk factor for the exacerbation of PAVF symptoms [5, 6]. PAVF patients often lack typical clinical presentations during pregnancy and are usually diagnosed upon the development of severe respiratory or neurological complications, including massive hemoptysis, hemothorax, and ischemic stroke. The mortality rate of PAVF is 1% in pregnant patients with good prenatal assessment [7]. Therefore, early diagnosis and management are critical for improving the prognosis of PAVF patients. The present study reports a rare case of gestational PAVF presented with transient hypoxemia and cerebral infarction. In addition, a literature review of PAVF during pregnancy is presented to provide a reference for the diagnosis and management of these patients.

## Case presentation

 A 29-year-old G2P1 pregnant woman at 34 weeks’ gestation was diagnosed with stable placenta accreta during a routine examination at the obstetric outpatient clinic of West China Second University Hospital, Sichuan University. The patient experienced dyspnea as she walked from the second floor to the outpatient clinic. Her pulse oximetry revealed an oxygen saturation level of 90%, which fluctuated between 90% and 95% during continued monitoring. After about 15 min of rest without oxygen inhalation, the patient experienced resolution of dyspnea, and her oxygen saturation level returned to 99%. The patient complained of progressively worsening dyspnea in the past month, which resolved spontaneously after resting without head elevation. The patient did not experience coughing, hemoptysis, nocturnal dyspnea, or other discomfort. Cardiopulmonary auscultation was unremarkable. The patient had a cesarean Sect. 3 years ago and had no underlying illnesses such as hypertension and diabetes. The patient’s physical examination was normal; thus, no further examinations or treatments were provided.

The patient was admitted to the Obstetrics Department of our hospital at 38 weeks of gestation for a cesarean section. After routine post-admission examinations, the patient underwent bilateral internal iliac artery balloon placement, followed by cesarean section under spinal-epidural anesthesia. The patient was slightly nervous before surgery but had normal vital signs and oxygen saturation. Epidural anesthesia (from T6 to S5) was successfully administered. One live infant was delivered successfully with clear amniotic fluid. However, the patient’s oxygen saturation gradually declined to 89% after delivery, which increased to 100% after administering 2 L/min of oxygen via a nasal cannula. The patient had no chest tightness, chest pain, shortness of breath, coughs, or remarkable fluctuations in heart rate and blood pressure. Auscultation revealed clear breath sounds without dry or wet rales and normal heart sounds. Skin mucosa was unremarkable. After the complete removal of the placenta and membranes, the patient exhibited poor uterine contraction and ruptured blood vessels in the lower uterine segment, resulting in substantial hemorrhage. Uterine massage and intramuscular injection of carboprost tromethamine were immediately provided to enhance uterine contraction. Emergency arterial blood gas analysis showed a PaO_2_ of 50 mmHg and Hb of 105 g/L with normal electrolytes. The uterine contraction improved and the bleeding was under control quickly. Coagulation, N-terminal brain natriuretic peptide level, and troponin level were unremarkable. D-dimer was 1.81 mg/L. Bedside electrocardiograph, echocardiogram, and vascular ultrasound of the lower extremities were normal. There were no drastic fluctuations in the patient’s heart rate, blood pressure, and respiration during the cesarean section. The operation lasted approximately 1.5 h with an estimated blood loss of 1000 mL. No blood transfusion was required.

**Fig. 1:**
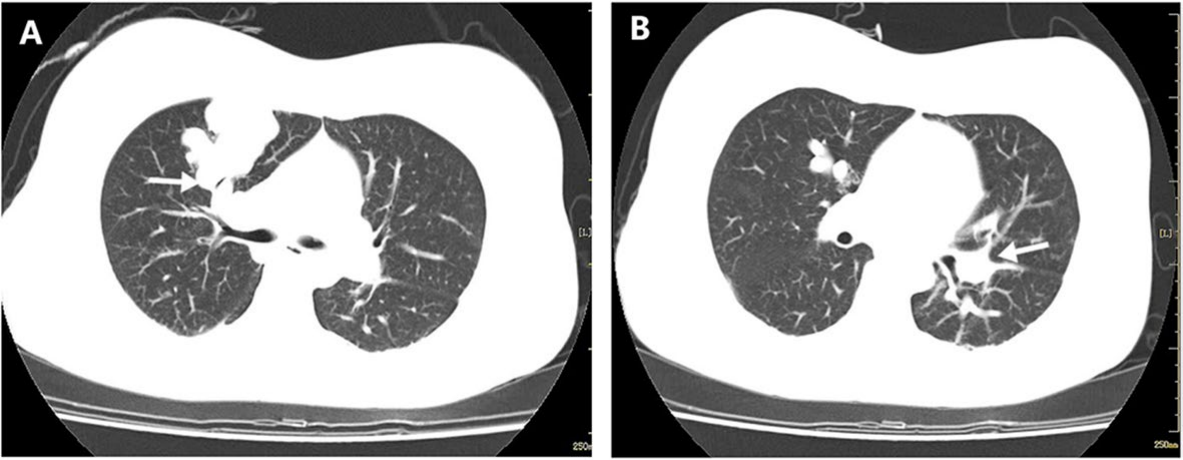
CTPA demonstrates multiple vascular malformations in both lungs. **A** In the anterior segment of the superior lobe of the right lung, the thickened right superior pulmonary artery is the feeding artery, and the right superior pulmonary vein is the drainage vein, with apparent signs of PAVF. **B** In the posterior basal segment of the lower lobe of the left lung, signs of PAVF are observed

The patient was transferred to the intensive care unit after surgery and had an oxygen saturation level of 99% with oxygen administration (2 L/min by nasal cannula). On day 1, after surgery, the patient was conscious without discomfort. Without oxygen administration, she had a 97% oxygen saturation level and began ambulatory activities. On day 3, after surgery, the patient exhibited slurred speech during ambulation, accompanied by right limb myasthenia. An emergency head magnetic resonance imaging revealed cerebral infarction in the left basal ganglia. The patient was subsequently referred for chest and abdominal computed tomography angiography, heart ultrasound, and transcranial Doppler ultrasound to identify the underlying cause of these symptoms. Computed tomography pulmonary arteriography (CTPA) revealed bilateral multiple pulmonary vascular malformations (Fig. 1). The patient’s symptoms significantly improved after antiplatelet and anticoagulant therapy. After one month of treatment, the patient was conscious and articulate, with normal muscle strength in her right limb. She was then transferred to the Department of Thoracic Surgery for percutaneous embolization of PAVF. During the telephone follow-up 6 months after surgery, the patient reported no discomfort or adverse impacts on her quality of life. Her child was also healthy.

## Discussion

PAVF in pregnancy is a rare and potentially fatal condition with limited experience in its identification and management. In this case study, the patient suffered a cerebral infarction three days after giving birth. She was diagnosed with PAVF, which might be the cause of cerebral infarction. Gestational conditions, postpartum hemorrhage, dilated and tortuous vessels near the arteriovenous fistula and poor circulation further increase the risk of thrombosis in PAVF patients [8]. Despite the active postoperative application of lower extremity pneumatic pump therapy and anticoagulant to prevent venous thrombosis, the patient still developed thrombosis. Furthermore, the thrombus flowed into pulmonary vein through a fistula and circulated to the cerebral vessels leading to the occurrence of cerebral infarction. A transient decrease in pulse oxygen saturation of unknown causes was the only clinical presentation of the patient in the late stage of pregnancy and after delivery, possibly as a result of the significant increase in blood flow through the right-to-left shunt in the lungs due to rapid changes in hemodynamics [6]. With the regulation of the distribution of blood circulation system, the right-to-left shunt decreased, and the oxygen saturation returned to normal.

Our case highlights the following main points. PAVF should not be neglected in pregnant women with transient hypoxemia and cerebral infarction. A transient decrease in pulse oxygen saturation that cannot be explained by common clinical causes might be an early warning sign of this medical condition.

Most patients show no typical symptoms, making early identification difficult [9]. We searched the Web of Science and PubMed databases without language restriction to identify case reports or case series published up to 30 June 2023 using the following terms: “pulmonary arteriovenous fistula or pulmonary arteriovenous malformation” and “pregnancy.” Twenty-nine cases of PAVF diagnosed during pregnancy were retrieved [3, 6, 10–36]. The patients’ characteristics and treatments are presented in Table 1. The gestational age of the pregnant patients in these case reports ranged from 7 to 39 weeks. There were 27 cases of PAVF in the second and third trimester. There were 20 cases with complications as the initial presentation, including 20 cases of hemothorax, and one case of cerebral infarction and deep venous thrombosis. One patient died due to severe respiratory failure. Dyspnea or hypoxemia was the most prominent early presentation in the remaining 9 cases who had no complications during pregnancy, consistent with that in general PAVF patients [37]. However, dyspnea, a non-specific complaint during the second and third trimester, is a common symptom of pregnancy, thus making it difficult to diagnose [18]. In contrast, hypoxemia may help in early identification of PAVF. The recurrent transient drop in oxygen saturation may be one of the clinical manifestations in pregnant women with PAVF and should therefore be closely monitored [38].

**Table 1:** 29 cases of PAVF diagnosed during pregnancy before 2023

Authors	Age(year)	The GA of events(weeks)	Symptoms	Complications	Comorbidity	Therapy	Date of delivery	Delivery mode	Maternal and fetal outcomes
Gammon RB et al. 1990 [10]	27	24	Chest pain, dyspnea and hemoptysis	Hypoxemia, hemothorax	No	Diuresis, transcatheter embolization	30	VD	Recovered well. A healthy infant
Chanatry BJ et al. 1992 [11]	23	35	Chest pain, dyspnea	Hypoxemia, hemothorax, hypertension	No	Thoracic tube drainage, cesarean section at 32 weeks, excision of diseased lung tissue	--	CS	Recovered well. A healthy infant
Laroche CM et al. 1992 [12]	37	29	Dyspnea, chest pain	Hemothorax, shock	HHT	Emergency lobectomy	37	CS	Recovered well. A healthy infant
Bevelaqua FA et al. 1992 [13]	24	25	Chest pain, dyspnea	hemothorax	No	Transcatheter embolization	Full term	VD	Recovered well. A healthy infant
Baumgardner DJ et al. 1993 [14]	25	23	Dyspnea	hypoxemia	HHT	Oxygen therapy, embolization.	36	VD	Recovered well. A healthy infant
Shovlin CL et al. 1995 [15]	17	16	Dyspnea	No	HHT	Monitored during pregnancy, postpartum embolization	Full term	CS	Recovered well. A healthy infant
Wilmshurst P et al. 1996 [16]	30	7	Dyspnea	Symptomatic exercise-induced hypoxemia	No	Oxygen therapy	Full term	VD	Recovered well. A healthy infant
Adegboyega PA et al. 1996 [17]	33	29	Chest pain	Hypoxemia, hemothorax	No	Oxygen therapy	Full term	CS	Recovered well. A healthy infant
Esplin MS et al. 1997 [18]	36	24	Dyspnea	Hemothorax	No	Transcatheter embolization, segmentectomy	36	CS	Recovered. A healthy infant
Gershon AS et al. 2001 [6]	28	29	Chest pain	Hemothorax	HHT	Oxygen therapy, chest tube drainage, transcatheter embolization	Full term	CS	Recovered. A healthy infant
Byung Ho Lee et al. 2002 [19]	38	28	Dyspnea	Hemothorax, hypoxemia	HHT	Transcatheter embolization	--	CS	Recovered. A healthy infant
Wong AS et al. 2006 [20]	33	31	Progressive dyspnea for 10 days, sudden chest pain	Hypoxemia, hemothorax	No	Thoracoscopic surgery, chest tube drainage	Precipitous preterm labor	VD	Recovered. The baby was ventilated in the neonatal ICU for 1 day because of respiratory distress syndrome
Zhao Y et al. 2010 [21]	34	22	Dyspnea	Hemothorax, hypoxemia	No	Surgery	Full term	CS	Recovered. A healthy infant
Anin SR et al. 2013 [22]	28	18	No	Mild low pulse oxygen saturation	No	Transcatheter embolization	Full term	VD	Recovered. A healthy infant
Di Crescenzo V et al. 2015 [23]	19	34	Dyspnea, chest pain	Hemothorax	No	Decortication for lung re-expansion and lung wedge resection	34	CS	Recovered. A healthy infant
Raiya S, et al. 2017 [24]	25	23	Sudden chest pain, dyspnea, cough	Hemothorax	HHT	Pleural aspiration, coil embolization	Full term	--	underwent transcatheter embolotherapy successfully in the second trimester. A healthy infant
Tajima H, et al. 2018 [25]	39	7	Incomplete motor, paralysis of the left upper and lower extremities	Pulmonary embolism, deep vein thrombosis, hemorrhagic cerebral infarction	HHT	A temporary inferior vena cava filter was implanted and removed after abortion. After rehabilitation with anticoagulant therapy, transcatheter embolization was performed.	7	abortion	Recovered
Texier C et al. 2018 [26]		26	Chest pain	Hemothorax	HHT	Transfusion, surgery	40	VD	Recovered. A healthy infant
Md Noh MSF et al. 2018 [27]	30	20	Dyspnea	Hemothorax	HHT	Coil embolization	20	CS	Recovered. Intrauterine fetal death
Wang HC et al. 2018 [28]	32	31	Sudden-onset dyspnea and backache	Hemothorax	No	Thoracoscopic surgery	31	CS	Recovered. A healthy infant
Borovac-Pinheiro A et al. 2019 [29]	31	14	Dyspnea	Hypoxemia	HHT	Oxygen therapy, transcatheter embolization	34	CS	A limited life. A healthy infant
Klein M et al. 2019 [30]	28	28	Hemoptysis	No	No	Treated with catheter-assisted coiling	38	CS	Recovered. A healthy infant
Di Guardo F et al. 2019 [31]	32	39	Dyspnea, then chest pain	Hemothorax	No	Transcatheter embolotherapy	39	CS	Recovered. A healthy infant
Naito J et al. 2020 [32]	34	28	Dyspnea, then chest pain	Hypoxemia	No	Thoracoscopic to open conversions	38	CS	Recovered. A healthy infant
Liu S et al. 2022 [3]	43	36 + 4	Dyspnea, then hemoptysis	No	HHT, PA, CHD: ASD	Termination	36 + 6	CS	Recovered. A healthy infant
van den Bulck M et al. 2022 [33]	25	34	Chest pain	Hemothorax	HHT	Chest tube drainage, termination and Transcatheter embolotherapy	34	CS	Recovered. A healthy infant
Zamaniyan M et al. 2022 [34]	34	20	Dyspnea, epigastric pain	Hemothorax	--	thoracotomy	20	CS	Dead fetus
Lukic A et al. 2023 [35]	26	20	Chest pain	Hemothorax	No	Chest tube drainage, surgery	38 + 4	CS	Recovered. A healthy infant
Robinson TJ et al. 2023 [36]	34	26	Abdominal pain	Hemothorax	No	Chest tube drainage, transcatheter embolotherapy	39	CS	Recovered. A healthy infant
Present case	29	34	Dyspnea	Transient hypoxemia, **c**erebral infarction	No	Oxygen therapy, Transcatheter embolotherapy	38	CS	Recovered. A healthy infant

*GA* gestational age; *CS* caesarean section; *VD* vaginal delivery; *CHD* *ASD* Congenital Heart Disease: Atrial Septal Defect; *PA* pulmonary hypertension; --: not mentioned in the literature.

Arterial blood gas analysis remains ideal to detect hypoxemia. If oxygen desaturation cannot be explained by common causes, PAVF should be considered as it is the most common cause of hypoxemia induced by extracardiac shunt. Large PAVF can generally be identified by plain or contrast-enhanced computed tomography scans [39]. Once identified, transthoracic contrast echocardiography can be performed to further examine the fistula [40]. Chest magnetic resonance imaging can also be a safer alternative for the fetus. CTPA or pulmonary arteriography can clearly show the site of PAVF and the number of supplying arteries. CTPA is the preferred diagnostic angiography approach due to its non-invasiveness. Once PAVF is confirmed, the physician should exclude HHT and assess whether vascular malformations exist at other sites, such as the heart, liver, and brain [41], because some patients may have HHT [7]. In our case, the patient had no history of frequent nasal bleeding, no personal or family history of telangiectasia, and no vascular malformations in other sites and was thus unlikely to be comorbid with HHT. Genetic testing was recommended to the patient for a definite diagnosis, but she refused due to financial constraints.

Currently, treatments for PAVF are decided based on clinical judgments, case reports in the literature, and consultations in a multidisciplinary team [35]. It has been reported that treatments should always be provided to patients with PAVF before pregnancy, regardless of the presence or absence of clinical symptoms, to avoid the development of potentially fatal complications during pregnancy [42]. For those with proper cardiopulmonary function, regular arterial blood gas monitoring and chest x-ray or computed tomography examinations should be performed to evaluate the progression of PAVF. Furthermore, doctors should carry out a dynamic assessment of thrombotic risk factors and take steps to prevent thrombosis [7]. PAVF patients are prone to severe complications during the second and third trimesters of pregnancy, childbirth, and the early postpartum period. Therefore, monitoring during these periods needs to be intensified. The approaches for delivery and anesthesia should be individualized. The endovascular technique of embolization has been recently accepted worldwide as mainstay of treatment and should be performed at time of diagnosis or when the following criteria are satisfied: progressive enlargement of a detected PAVF, symptomatic hypoxemia, or serious complications [43]. It is safe and effective after 16 weeks of gestation [6, 44]. Surgery during pregnancy poses a significant clinical challenge as it can lead to atelectasis, pulmonary edema, and premature birth. The effect of pregnancy on PAVF recanalization is currently unclear, and postpartum patients should undergo long-term follow-ups [45].

## Conclusions

This case emphasizes that PAVF should be suspected if a pregnant patient presents with transient hypoxemia and cerebral infarction. And a transient decrease in pulse oxygen saturation that cannot be explained by common clinical causes can be an early indication of this disease. The physician should be alert to complications of pulmonary arteriovenous fistula during pregnancy. Early diagnosis and multidisciplinary management could improve the prognosis.

## Data Availability

The data sets used and analyzed during the current study are available from the corresponding author upon reasonable request.

## Abbreviations

PAVF: Pulmonary arteriovenous fistula
CTPA: Computed tomography pulmonary arteriography
HHT: Hereditary hemorrhagic telangiectasia
