# Correction: Attention to pulmonary arteriovenous fistula in a case of transient hypoxemia and cerebral infarction during pregnancy: a case report and literature review

**DOI:** 10.1186/s12884-023-06007-4

**Published:** 2023-09-21

**Authors:** Lijuan Shu, Linli Luo, Yunxia Zuo

**Affiliations:** 1 Department of Anesthesiology, West China Hospital, Sichuan University, Chengdu, 610041 Sichuan P.R. China; 2 West China Hospital, The Research Units of West China (2018RU012) ‑ Chinese Academy of Medical Sciences, Sichuan University, Chengdu, 610041 Sichuan P.R. China; 3 Key Laboratory of Birth Defects and Related Diseases of Women and Children, Sichuan University, Ministry of Education, Chengdu, 610041 Sichuan P.R. China; 4 Department of Obstetrics and Gynecology Intensive Care Unit, West China Second University Hospital, Sichuan University, Chengdu, 610041 Sichuan P.R. China; 5 Department of Anesthesiology, West China Second University Hospital, Sichuan University, Chengdu, 610041 Sichuan P.R. China

**Correction: BMC Pregnancy Childbirth 23, 626 (2023)**

10.1186/s12884-023-05946-2

Following publication of the original article [1], the authors reported an error in the affiliations. Affiliations 4 and 5 are need to be swapped.

The author group has been updated above and the original article has been corrected.
